# Is area-level socioeconomic deprivation associated with mortality due to circulatory system diseases in Poland?

**DOI:** 10.1186/s12889-022-14914-y

**Published:** 2023-01-03

**Authors:** Jacek Jamiołkowski, Agnieszka Genowska, Andrzej Pająk

**Affiliations:** 1 Department of Population Medicine and Lifestyle Diseases Prevention, Medical University of Bialystok, Białystok, Poland; 2 Department of Public Health, Medical University of Bialystok, Białystok, Poland; 3 Department of Epidemiology and Population Studies, Jagiellonian University Medical College, Kraków, Poland

**Keywords:** Cardiovascular disease, Deprivation, Smoking, Body mass index, Urbanization, Health inequalities

## Abstract

**Background:**

Socioeconomic deprivation (SED) is known to influence cardiovascular health. However, studies analyzing the relationship between deprivation and circulatory system diseases (CSD) in Central and Eastern Europe are limited. This study aimed to assess the relationship between SED and mortality due to CSD at a population level in 66 sub-regions of Poland.

**Methods:**

The 2010–2014 data regarding mortality and SED components were obtained from the Central Statistical Office. An area-based SED index was calculated based on the higher education rates, employment structure, wages, unemployment, and poverty. The dynamics of changes in mortality due to CSD was expressed by the number of deaths prevented or postponed (DPP) in terciles of the SED index. The associations between the mortality from CSD and SED index were analyzed using multivariate Poisson regression models and generalized estimating equations.

**Results:**

Among men, the percentage of DPP in 2014 was 13.1% for CSD, 23.4% for ischemic heart disease (IHD), and 21.4% for cerebrovascular diseases (CeVD). In the case of women, the proportion of DPP was 12.8, 25.6, and 21.6%, respectively. More deprived sub-regions experienced a greater decrease in CSD-related mortality than less deprived sub-regions. The disparity in mortality reduction between more deprived and less deprived sub-regions was even more pronounced for women. After adjusting for smoking prevalence, average BMI, population density, and changes in mortality over time, it was found that the SED index over the 2010–2014 time period was significantly associated with CSD- and IHD-related mortality for men (respectively 5.3 and 19.5% expected mortality increase per 1-unit increase of SED index), and with IHD- and CeVD-related mortality for women (respectively 30.3 and 23.0% expected mortality increase per 1-unit increase of SED index).

**Conclusions:**

Significant differences in mortality changes due to CSD in Poland could be observed in relation to socioeconomic deprivation, resulting in reduced health inequalities. To reduce CSD mortality, more comprehensive preventive measures, including approaches addressing the socioeconomic factors, mainly poverty, education and employment, are needed, particularly in less urbanized areas.

**Supplementary Information:**

The online version contains supplementary material available at 10.1186/s12889-022-14914-y.

## Background

Circulatory system diseases (CSD), which originate from atherosclerotic disease and include both the ischemic heart disease (IHD) and cerebrovascular diseases (CeVD), have been reported for many years the most common causes of death in the developed countries. In nonfatal cases, long-term treatment is required, which limits an individual’s capacity to work and consequently incurs a high cost in terms of both treatment and loss of professional productivity, particularly in those over 50 years of age [1, 2].

The burden of CSD is unequally distributed across Europe, with higher mortality in the central and eastern regions [3, 4]. This higher burden reported in post-communist countries has been somewhat inherited due to several historical reasons associated with the socioeconomic system of that time. Central and Eastern European countries are characterized by a lower gross domestic product per capita, and thereby, a lower expenditure on health care, and also limited access to health care and prevention services. Furthermore, the health care gap between the Eastern and Western populations may result from, or could be explained by, lifestyle and differences in socioeconomic influences [5]. The difference in mortality due to CSD depends on socioeconomic position (SEP), which is determined mainly by education, type of occupation, and income. This indicates the need to include SEP in the risk assessment of CSD along with the classical factors: smoking, high blood pressure, hypercholesterolemia, overweight and obesity, sedentary lifestyle and diabetes [6, 7]. Low SEP may contribute to stress-related biological risk factors associated with the development of cardiovascular diseases (hypertension, proinflammatory cytokines, diabetes, obesity) [8].

Furthermore, it may lead to poorer health as a result of harmful health behaviors, such as tobacco smoking, an unhealthy diet, and lower physical activity [9, 10]. In addition to individual factors, CSD-related mortality could be linked with socioeconomic deprivation (SED) at the area level [11–15]. Deprivation is a state of observable and demonstrable disadvantages, which can be of social or material nature, compared to the local community or the wider society to which an individual, family, or group belongs [16]. The area-related deprivation serves as a proxy for the SEP of the people who live in those regions, and characterizes the living environment which can influence an individual’s prospects for education, profession, income, or access to resources, and hence, health outcomes [7, 17]. Furthermore, neighborhood factors could have an impact on health through a lack of investments or inequality in resource redistribution in society. Moreover, chronic exposure to psychosocial stress, caused by poverty areas, is linked to the development of multisystemic adverse biological changes, i.e., allostatic burden, which results in cardiovascular diseases [18]. Studies indicate that factors associated with area SED could significantly affect short-term survival, while individual factors may considerably impact long-term survival [12].

The majority of information on the relationship between SED and cardiovascular disease-related mortality comes from studies conducted in Western Europe and the United States [13–15, 19, 20]. At the same time, much fewer data are available from the Central and Eastern European countries [21, 22]. Recent studies conducted in Poland, Russia, and the Czech Republic have confirmed a strong association between the risk of death from CSD and psycho-socioeconomic factors [23, 24]. However, there is a need for a more extensive assessment of the factors determining health inequalities among people in the Central European countries, including Poland. The political transformation that occurred in 1989 in Poland led to significant changes in educational and occupational structures [25, 26]. Despite the considerable increase in SEP, some groups adapted to the new market economy at a slower rate. The initial post-transition period in the 1990s brought adverse consequences to those entering the labor market. In particular, young people with low levels of education, whose poorer material status contributed to poorer health outcomes, were less resistant to these changes [27]. Furthermore, economic changes have clearly reduced employment in the heavy industry and national farm holdings [27, 28], leading to economic and social inequalities in the area of residence, i.e., impoverishment and social exclusion, and consequently excessive alcohol consumption due to changes in availability and affordability [25, 29]. These educational and economic inequalities that occurred in the early years of the transformation may now be affecting CSD mortality, including premature mortality under the age of 65 [30]. Thus far, analyses have primarily focused on changes in CSD mortality caused by changes in exposure to some of classical risk factors (cholesterol level, blood pressure), lifestyle changes, and health care services improvement [31, 32]. No study has investigated the socioeconomic factors at the population level. Territorial variations in CSD mortality in Poland may likely be explained by SED differences and their changes over time.

This study aimed to assess the relationship between SED and mortality due to CSD at a population level in 66 sub-regions of Poland.

## Methods

An ecological study was performed using the data on SED, lifestyle, urbanization, and mortality due to CSD collected from 66 administrative sub-regions of Poland. The sub-regions were defined according to the Nomenclature des Unités Territoriales Statistiques (NUTS-3) in 2006 [33]. The mean size of the sub-regions was 4738 km^2^, with a wide range of variation between eight urban-type sub-regions (from 262 to 517 km^2^) and 58 remaining sub-regions (from 878 to 12,090 km^2^). Average number of population per sub-region (in year 2010) was 583,786, and ranged from 279,491 to 1,700,112. Descriptive statistics for the sub-regions were weighted to the population of sub-regions using weights as follows:

$${W}_x=\frac{population_x\bullet n}{population_{total}},$$

where:

*population*_*x*_ is the population of sub-region x,

*population*_*total*_ is the total population of Poland, and.

*n* is the number of sub-regions.

The sum of weights for all sub-regions equals the number of sub-regions.

Annual data for mortality, all components of the SED index, and population density in 2010–2014 were obtained from the Central Statistical Office. In some analyses - where explicitly stated - the data were averaged over this period. Otherwise - like in longitudinal statistical models, separate data for each year were used. Information on average population BMI and proportion of smokers come from the panel Social Diagnosis Survey conducted repeatedly since 2000 on the same sample of households [34]. For these variables, data from the 2011 survey was used and treated as static for the whole 5-year period between 2010 and 2014.

### Mortality

Data on deaths during 2010–2014 was used to analyze mortality. According to the International Statistical Classification of Diseases and Related Health Problems, Tenth Revision (ICD-10), the causes for the deaths were encoded as CSD (codes: I00–I99), including IHD (I20–I25) and CeVD (I60–I69).

The age-standardized number of deaths was used for statistical analyses. A direct standardization method was utilized for the analyses, considering the demographic structure in 5-year age groups (from 15 to 19 years to 80–84 years, and above 84 years old) separately for each of the 66 sub-regions of Poland [35]. European Standard Population – 2013 edition was assumed as the standard population, and standardized mortality coefficients were determined accordingly [36]. The coefficients of standardized mortality were calculated as 95% confidence intervals (CIs).

The dynamics of changes in mortality rates in 2010–2014 were assessed by calculating the deaths prevented or postponed (DPP) index [37]. The index value was expressed in percentage and estimated as the difference between the expected number of deaths in 2014, assuming that the mortality rate in 2014 was the same as in 2010, and the actual number of deaths in 2014 relative to the expected number of deaths in 2014:

$$DPP=\frac{{deaths\ expected}_{2014}-{deaths\ observed}_{2014}}{{deaths\ expected}_{2014}} \bullet 100\%$$

### Area-level SED index

Five SED indicators were selected from the available variables in the system of routine statistical records from the Central Statistical Office. The selected indicators of SED were as follows: people with higher education, people employed in industry and construction, average monthly salary, unemployment rate, and people on social support due to poverty. These variables were chosen as they showed a strong correlation with all-cause mortality [38]. According to published literature data, the SED variables are potential predictors of the geographical distribution of CSD mortality [39–44]. Education not only increases the awareness of beneficial health behaviors through medical and prophylactic care and treatment recommendations, but is also one of the essential determinants of employment [39, 40]. The professional class, characterized by a balance between effort and reward, is associated with health. Similarly, people employed in industry and construction are manual workers and exhibit worse health outcomes [41]. Income determines an individual’s access to better-quality goods, services and housing to stay healthy [42]. Thus, unemployment is linked with disease development due to reduced access to medical care and screening programs resulting from the lack of health insurance [43]. Poverty associated with a lack of social support leads to chronic stress and adverse health behaviors [18, 44]. To calculate the SED index, its component variables were standardized via linear transformation such that the expected value of the variables was equal to 0 and the standard deviation was equal to 1. In addition, the sign was reversed for destimulants (proportion of people with higher education and average monthly salary). Finally, the SED index was assumed to be the arithmetic mean of the transformed components and calculated from the averaged values of component variables separately for the years 2010–2014. Therefore, the proposed SED index might be considered the mean of Z-Scores of its components, taking into account inverted scales for destimulant variables. The calculated index values were used to explain the differences in mortality across sub-regions of Poland. In addition, based on the 5-year averaged values of the SED index, the 66 sub-regions were divided into three groups with equal number of areas and with lower and upper tercile of averaged SED index as cut-off points:

- the group with the lowest index values and thus the lowest deprivation level (less deprived),
- the middle group,
- the group with the highest index values (more deprived).

This classification was used to emphasize the inequalities in mortality for descriptive purposes.

### Other explanatory variables

Tobacco consumption and body mass index (BMI) were calculated for the 66 sub-regions based on the results of the Social Diagnosis Survey conducted in 2011. The survey involved 12,386 households selected based on the two-stage stratified sampling method—first at the voivodeship level, followed by sampling based on residential location (large cities, small cities, and villages) [34]. The study sample comprised 36,753 persons aged 16 years and over. The participation rate of the study was 72% (i.e., 26,453 persons were finally examined). To eliminate the socioeconomic differences in urbanization between the sub-regions in the model, population density was included as a standardizing factor [19, 45].

### Statistical analysis

The relationship between mortality and the SED index was determined using the multivariate Poisson regression model for count data. As this model reflected the mortality coefficient per 100,000 residents rather than the number of deaths, the age-standardized number of deaths in individual sub-regions was taken as a dependent variable, with the number of people in sub-regions serving as the offset. The generalized linear models with Poisson distribution as probability distribution and logarithm as a link function were used for calculations.

Due to repeated measurements in the same statistical units (mortality in sub-regions in subsequent years during 2010–2014), generalized estimating equations (GEE) were used to obtain generalized linear models for the correlated data [46]. An exchangeable structure was assumed for the working correlation matrix in GEE models.

The Poisson models were presented with exponentially transformed regression coefficients. Therefore, they can be interpreted as the expected relative change of the dependent variable (number of deaths standardized for the age) calculated for a one-unit increase of the independent variable, i.e., relative risk. Therefore, the reported coefficients can also be interpreted as a relative change in mortality coefficients, and not just death numbers. Furthermore, 95% CIs were calculated for the coefficients of the regression model and their *p*-values of corresponding Wald’s tests.

Variance Inflation Factors were calculated to check the presence of a potential multicollinearity problem in multivariable models. In no case did the VIF exceed the value of 4 taken as a threshold, and the maximum value observed was 3.84.

The regression models developed for the study included three causes of death (CSD, IHD, CeVD) for men and women. Furthermore, as independent variables, calendar year (for assessing the time effect on the dependent variable) and the aforementioned index - level of socioeconomic deprivation in the sub-regions - were included in the models. In addition, the models included lifestyle factors, such as mean BMI and the average percent of smokers in the sub-region (for the female and male population, respectively) and population density in the sub-region using logistic transformation at base 2.

Simple, univariate relationships between the SED index and mortality have been assessed with Spearman’s nonparametric correlation coefficients.

Statistical calculations were performed in the IBM® SPSS® Statistics for Windows statistical package, version 20.0 (IBM Corporation, Armonk, NY, USA). The level of statistical significance was assumed at *α* = 0.05.

## Results

During 2010–2014, a total of 868,418 deaths due to CSD were recorded in Poland, which included 405,235 deaths among men (46.7%). Of the overall death number, the deaths due to CSD in men accounted for 40.6%, while the death proportion in women reached 51.2%. Among CSD, IHD (32.0%) and CeVD (24.6%) were the leading causes of death.

The descriptive statistics for socioeconomic variables, lifestyle, and urbanization, by three sub-groups of Polish sub-regions (classified based on the SED index values), are presented in Table 1. The mean values of the index variables in sub-groups reflect their role as stimulants or destimulants—in the sub-regions characterized by the highest SED index (a more deprived), high values were attained by stimulants and low values by destimulants. Therefore, this group of sub-regions had the highest percentage of unemployment rate (18.4%), percentage of people who are on social support due to poverty (11.1%) and people employed in industry and construction (31.1.%), as well as the lowest percentage of people with university education (12.9%) and people with average monthly salary (3108 PLN). Opposite characteristics occurred in the sub-regions with the lowest SED index values (less deprived), which had the lowest unemployment rate (8.9%) and the lowest percentage of people on social support due to poverty (5.3%). On the other hand, these sub-regions had the highest percentage of people with a university education (22.5%) and the highest average monthly salary (3916 PLN). The precise distributions of SED components are shown on the maps presented in Supplemental Fig. 1.

**Table 1:** Descriptive statistics for SED, lifestyle characteristics, and urbanization in 66 sub-regions of Poland during the period 2010–2014 (averaged annual data) by categories of SED index weighted for the size of sub-region population

Index of socioeconomic deprivation (tercile)	1st tercile (less deprived)	2nd tercile (middle)	3rd tercile (more deprived)
SED index range	−2.66 to − 0.07	−0.07 to 0.33	0.34 to 1.15
*SED INDEX and its components*			
SED index	−0.92 ± 0.82 (−1.64/− 0.58/− 0.27)	0.17 ± 0.10 (0.10/0.18/0.26)	0.63 ± 0.28 (0.42/0.50/0.88)
University education [%]	22.48 ± 7.66 (17.14/20.36/28.49)	13.62 ± 1.75 (12.60/13.38/14.12)	12.92 ± 1.68 (12.00/12.69/14.07)
Employed in industry and construction [%]	27.54 ± 9.41 (20.54/25.24/34.72)	27.30 ± 10.04 (18.47/27.16/35.20)	31.05 ± 5.33 (27.57/33.20/34.83)
Average salary [PLN]	3916 ± 550 (3463/3852/4260)	3173 ± 207 (3048/3135/3237)	3108 ± 156 (2972/3113/3227)
Unemployment rate [%]	8.93 ± 3.34 (5.54/9.49/11.26)	13.90 ± 2.33 (13.14/14.33/15.17)	18.37 ± 3.66 (16.09/18.56/20.49)
People on social support due to poverty [%]	5.30 ± 1.70 (3.44/5.33/6.35)	9.10 ± 2.16 (7.24/9.44/10.56)	11.10 ± 2.25 (8.85/11.62/13.33)
*Lifestyle indicators*			
Smoking (men) [%]	32.3 ± 4.4 (28.6/32.9/35.3)	31.5 ± 4.9 (28.4/30.8/34.1)	35.8 ± 6.3 (31.5/35.9/41.8)
Smoking (women) [%]	22.4 ± 4.4 (20.5/22.8/26.2)	16.9 ± 4.1 (14.4/16.2/19.4)	20.9 ± 5.1 (18.4/20.4/25.5)
BMI (men) [kg/m^2^]	26.4 ± 0.4 (26.0/26.3/26.8)	26.4 ± 0.3 (26.2/26.3/26.5)	26.6 ± 0.4 (26.4/26.6/26.8)
BMI (women) [kg/m^2^]	25.0 ± 0.4 (24.6/25.1/25.3)	25.4 ± 0.3 (25.1/25.3/25.6)	25.4 ± 0.4 (25.1/25.4/25.6)
Urbanization			
Population density [n/km^2^]	1124.8 ± 1167.4 (171.4/395.6/2141.9)	127.3 ± 79.7 (78.0/97.7/163.2)	89.5 ± 30.4 (62.3/88.6/106.0)

Values are presented as mean ± standard deviation (first quartile/median/third quartile)

Abbreviation: SED–socioeconomic deprivation, BMI—body mass index

A substantial difference in population density was noted between the three groups of sub-regions. In the more deprived sub-regions, it was on average 89 people/km^2^. In contrast in the less deprived sub-regions, it was over 13 times higher—1125 people/km^2^, which indicates that low SED index values are mainly linked with highly urbanized areas. The percentage of smoking men was similar in all the three groups of sub-regions, ranging from 32.3 to 35.8%, and in women from 20.9 to 22.4%, whereas a higher proportion of smoking men was found in the tercile representing those more deprived, while for women the highest proportion of smokers was observed in less deprived group. No significant differences were found in nutritional behaviors. The mean BMI of male and female populations was very similar in all three groups of sub-regions (26.4–26.6 kg/m^2^ for males and 25.0–25.4 kg/m^2^ for females).

**Fig. 1:**
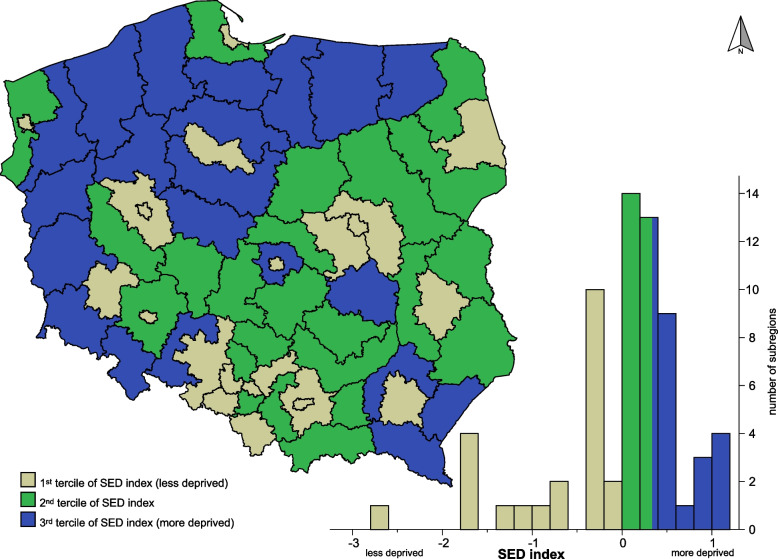
Geographic variation of mean socioeconomic deprivation index (66 sub-regions of Poland, averaged data over the years 2010–2014). Map created using IBM SPSS Statistics for Windows ver. 20.0 - https://www.ibm.com/products/spss-statistics

The territorial distribution of sub-regions by SED index is presented in Fig. 1. The less deprived sub-regions with the lowest SED index value primarily had large urban agglomerations.

The highest mortality due to CSD was in the more deprived sub-regions and the middle group, and the lowest in those less deprived. At the same time, it can be observed that the dynamics of change in mortality expressed as a percentage of DPP varied among deprivation-defined tercile groups of sub-regions. The percentage of avoided deaths increased significantly with an increase in the SED index (*p* < 0.001). In the case of men, the IHD-related mortality was the lowest in the more deprived sub-regions. In addition, an increase in the percentage of DPP was observed in these sub-regions (*p* < 0.001). The relationship between the deaths due to CeVD in men and the SED index was unambiguous. The highest CeVD-related mortality was observed in the sub-regions characterized by an average level of socioeconomic deprivation. However, a statistically significant linear increase in the percentage of DPP was observed in these sub-regions (*p* < 0.01) (Table 2).

**Table 2:** Age-standardized mortality rates from CSD and proportion of DPP in men in 2010 and 2014 by the index of SED tercile groups

Index of SED	1st tercile (less deprived)		2nd tercile (middle)		3rd tercile (more deprived)		Total	
Calendar year	2010	2014	2010	2014	2010	2014	2010	2014
Population number	*N* = 6,177,556	*N* = 6,176,172	*N* = 4,981,098	*N* = 4,993,735	*N* = 4,491,158	*N* = 4,491,604	*N* = 15,649,812	*N* = 15,661,511
*Circulatory system diseases*								
SMR (95% CI)	867.80 (857.68–877.93)	768.16 (759.44–776.89)	1000.08 (987.59–1012.56)	867.96 (857.03–878.89)	1003.10 (989.06–1017.13)	858.13 (846.06–870.21)	944.81 (937.93–951.69)	822.42 (816.47–828.36)
Observed number of deaths	31,614	31,321	27,128	25,655	22,705	21,827	81,447	78,803
Expected number of deaths*		35,434		29,839		25,291		90,649
Number (percentage) of DPP relative to expected deaths		4113 (11.6%)		4184 (14.0%)		3464 (13.7%)		11,846 (13.1%)
Linear trend for DPP	*p* < 0.001							
*Ischemic heart disease*								
SMR (95% CI)	266.91 (261.32–272.50)	217.49 (212.84–222.14)	288.34 (281.65–295.03)	208.50 (203.21–213.80)	259.22 (252.13–266.30)	185.19 (179.65–190.72)	271.68 (268.01–275.35)	206.44 (203.48–209.40)
Observed number of deaths	10,240	9206	8291	6695	6342	5142	24,873	21,043
Expected number of deaths*		11,353		9067		7029		27,468
Number (percentage) of DPP relative to expected deaths		2147 (18.9%)		2372 (26.2%)		1887 (26.8%)		6425 (23.4%)
Linear trend for DPP	*p* < 0.001							
Cerebrovascular diseases								
SMR (95% CI)	156.82 (152.45–16,119)	123.73 (120.20–127.26)	199.50 (193.86–205.15)	156.16 (151.45–160.86)	173.50 (167.60–179.41)	136.00 (131.12–140.89)	174.77 (171.77–177.76)	136.96 (134.50–139.42)
Observed number of deaths	5722	5143	5149	4383	4457	3834	15,328	13,360
Expected number of deaths*		6389		5697		4896		16,991
Number (percentage) of DPP relative to expected deaths		1246 (19.5%)		1314 (23.1%)		1062 (21.7%)		3631 (21.4%)
Linear trend for DPP	*p* < 0.01							

Abbreviations: CSD —circulatory system diseases; DPP—deaths prevented or postponed; SED—socioeconomic deprivation; SMR—standardized mortality rate per 100,000 population; CI—confidence interval;

*Assuming that death rates by age groups from 2010 persist in 2014

The relationship between the SED index and CSD-related mortality in women was similar to that in men. The age-standardized CSD mortality was the lowest in the sub-regions with the lowest deprivation, while the highest occurred in the middle group sub-regions. The percentage of prevented deaths increased significantly with the SED index value (*p* < 0.001). Similarly, mortality due to IHD was the lowest in the more deprived sub-regions and the highest (in 2014) in the less deprived sub-regions. The percentage of DPP increased significantly (*p* < 0.001) with an increase in the SED index value. Similar to men, mortality due to CeVD in women was the highest in middle sub-regions and the lowest in less deprived sub-regions. No significant differences were observed in the percentage of prevented deaths (*p* = 0.11). The differences were found at the same level in all the three groups of sub-regions studied (Table 3).

**Table 3:** Age-standardized mortality rates from CSD and proportion of DPP in women in 2010 and 2014 by the index of SED tercile groups

Index of SED	1st tercile (less deprived)		2nd tercile (middle)		3rd tercile (more deprived)		Total	
Calendar year	2010	2014	2010	2014	2010	2014	2010	2014
Population number	*N* = 6,960,632	*N* = 6,972,552	*N* = 5,285,442	*N* = 5,303,620	*N* = 4,778,214	*N* = 4,776,768	*N* = 17,024,288	*N* = 17,052,940
*Circulatory system diseases*								
SMR (95% CI)	583.09 (577.17–589.02)	525.64 (520.35–530.92)	675.01 (667.70–682.31)	581.20 (574.79–587.62)	652.36 (644.44–660.27)	550.03 (543.18–556.87)	630.58 (626.59–634.57)	549.65 (546.14–553.15)
Observed number of deaths	35,893	36,652	31,330	30,194	25,255	24,060	92,478	90,906
Expected number of deaths*		39,876		33,396		30,998		104,307
Number (percentage) of DPP relative to expected deaths		3483 (8.7%)		5228 (15.7%)		4653 (15.0%)		13,401 (12.8%)
Linear trend for DPP	*p* < 0.001							
*Ischemic heart disease*								
SMR (95% CI)	140.78 (137.80–143.75)	114.80 (112.28–117.32)	154.05 (150.46–157.63)	106.72 (103.89–109.55)	131.79 (128.14–135.44)	90.60 (87.76–93.44)	142.63 (140.69–144.57)	105.88 (104.30–107.46)
Observed number of deaths	8693	7998	7167	5521	5099	3975	20,959	17,494
Expected number of deaths*		9802		7974		5734		23,523
Number (percentage) of DPP relative to expected deaths		1804 (18.4%)		2453 (30.8%)		1759 (30.7%)		6029 (25.6%)
Linear trend for DPP	*p* < 0.001							
*Cerebrovascular diseases*								
SMR (95% CI)	126.60 (123.79–129.42)	98.83 (96.48–101.17)	155.87 (152.27–159.46)	121.97 (118.94–124.99)	128.49 (124.91–132,07)	102.09 (99.07–105.11)	136.39 (134.50–138.28)	106.94 (105.36–108.53)
Observed number of deaths	7861	6875	7307	6310	5063	4458	20,231	17,643
Expected number of deaths*		8806		8049		5627		22,500
Number (percentage) of DPP relative to expected deaths		1931 (21.9%)		1739 (21.6%)		1169 (20.8%)		4857 (21.6%)
Linear trend for DPP	*p* = 0.11							

Abbreviations: CSD —circulatory system diseases; DPP—deaths prevented or postponed; SED—socioeconomic deprivation; SMR—standardized mortality rate per 100,000 population; CI—confidence interval;

*Assuming that death rates by age groups from 2010 persist in 2014

**Fig. 2:**
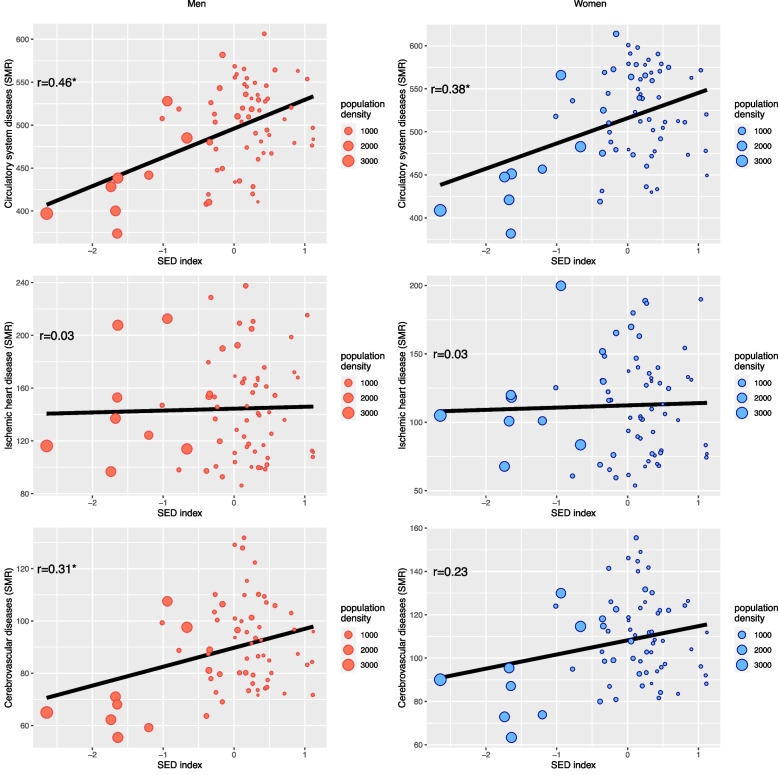
Scatterplots of CSD mortality (standardized mortality rates) and SED index with the additional indication of population density in 66 sub-regions of Poland (data averaged over the 2010–2014 period) * statistically significant correlations at *p* < 0.05

Univariate relationships between the SED index and age-standardized mortality in 66 sub-regions of Poland averaged over the 2010–2014 period are presented in Fig. 2. Statistically significant positive correlations were observed for CSD mortality both in males (r = 0.46) and females (r = 0.38) and also for CeVD in males (r = 0.31), however no significant correlations for IHD were observed.

As univariate analysis neglects other potentially important variables, the relationships between CSD mortality and time, SED, lifestyle, and population density are presented in Table 4. As the model is more detailed and adjusts for additional sources of variability, its results are not the same. The relationship between the SED and increased mortality was found for deaths due to CSD and IHD in men and deaths due to IHD and CeVD in women. For both women and men, a statistically significant decrease was observed in mortality due to CSD (2.9% for men and 2.8% for women annually), IHD (6.6% for men and 7.7% for women annually), and CeVD (6.1% for men and 6.6% for women annually). A higher mean BMI was associated with higher mortality due to CSD among women, but no significant relationship was observed between BMI and mortality due to IHD and CeVD. In the case of men, a relationship was noted between smoking and mortality due to CSD. Low SED index (by and large related to urban agglomerations) was found to be related to lower mortality. In comparison, a high population density was associated with greater IHD mortality in women and men and CeVD mortality only in women.

**Table 4:** Relation between mortality from CSD and SED index (as a continuous variable), lifestyle, and population density in 66 sub-regions of Poland in the period 2010–2014

Group diseases	Variables in the model	Men		Women	
		exp(β) with 95% CI	*p*	exp(β) with 95% CI	*p*
Circulatory system diseases (ICD-10: I00–I99)	Time [1-year increase]	**0.971 (0.967, 0.976)**	**0.000**	**0.972 (0.966, 0.978)**	**0.000**
	SED index [1-unit increase]	**1.053 (1.008, 1.100)**	**0.020**	1.047 (0.992, 1.106)	0.092
	BMI [1-kg/m^2^ increase]	1.018 (0.956, 1.084)	0.584	**1.076 (1.009, 1.148)**	**0.026**
	Smoking [1% increase]	**1.005 (1.002, 1.009)**	**0.004**	0.996 (0.991, 1.001)	0.111
	Population density [twofold increase]	0.995 (0.972, 1.017)	0.639	1.004 (0.978, 1.030)	0.776
Ischemic heart disease (ICD-10: I20–I25)	Time [1-year increase]	**0.934 (0.914, 0.955)**	**0.000**	**0.923 (0.891, 0.956)**	**0.000**
	SED index [1-unit increase]	**1.195 (1.005, 1.419)**	**0.043**	**1.303 (1.025, 1.657**)	**0.031**
	BMI [1-kg/m^2^ increase]	1.079 (0.895, 1.300)	0.426	0.994 (0.764, 1.293)	0.963
	Smoking [1% increase]	1.003 (0.990, 1.015)	0.698	0.994 (0.977, 1.012)	0.520
	Population density [twofold increase]	**1.094 (1.006, 1.189)**	**0.036**	**1.145 (1.018, 1.288)**	**0.024**
Cerebrovascular diseases (ICD-10: I60–I69)	Time [1-year increase]	**0.939 (0.930, 0.949)**	**0.000**	**0.934 (0.914, 0.955)**	**0.000**
	SED index [1-unit increase]	1.008 (0.921, 1.102)	0.869	**1.230 (1.025, 1.477)**	**0.026**
	BMI [1-kg/m^2^ increase]	1.090 (0.979, 1.213)	0.117	0.983 (0.809, 1.194)	0.862
	Smoking [1% increase]	1.006 (0.998, 1.014)	0.113	0.993 (0.977, 1.008)	0.342
	Population density [twofold increase]	0.967 (0.918, 1.019)	0.208	**1.107 (1.012, 1.211)**	**0.027**

CSD —circulatory system diseases; SED—socioeconomic deprivation

Separate models for each disease group by gender including the effects of time trend, SED index, BMI, prevalence of smoking, and population density. Due to data availability BMI and smoking were treated as static over the whole time period

Significant results are shown in bold

Abbreviations: exp.(β)—exponentiated coefficient of Poisson regression model, CI—confidence interval, ICD-10—International Statistical Classification of Diseases and Related Health Problems, Tenth Revision; BMI—body mass index

## Discussion

### Main findings

The study showed a considerable geographic variation in CSD mortality and SED in the 66 sub-regions of Poland. For both men and women, the largest mortality was found in the more deprived and middle group sub-regions, and the lowest in the less deprived sub-regions. However, the more deprived and middle group sub-regions benefited more from decreased CSD mortality in men and women. This indicates a reduction in health inequalities, which was also observed in other European countries [47, 48]. In women, the differences in the number of DPP between the SED groups were similar to that in men, except in the case of CeVD for which a nonsignificant trend was observed. The relationship between SED and CSD mortality was significant in men after adjusting for mean BMI, mean smoking rate, and population density. In women, a significant relationship was observed with mean BMI. However, the relationship between CSD mortality and SED was insignificant after adjusting for covariates, although the *β* coefficients were similar to that of men.

### Interpretation of results

Our study indicates that the variation of CSD mortality in Poland’s sub-regions can be explained at least partially by the differences in SED. The results comply with the studies in which synthetic SED indexes were used to assess the relationship between deprivation and mortality due to CSD, IHD and CeVD [13–15, 19–22, 49]. It has been suggested that deprivation significantly influences the mortality due to CSD in men, but not women [13, 50]. We did not show a significant relationship in women, however the effect of SED is similar in size to that in model for men, which may indicate insufficient statistical power of the study.

While the impact of lifestyle on cardiovascular health is well known, it should be noted that there are gender differences in behavioral risk factors. For example, smoking prevalence traditionally affected more men than women [51]. In our study, CSD mortality was shown to be associated with tobacco smoking only in men. This could reflect the higher smoking rates in the group of more deprived sub-regions compared with the less deprived sub-regions. According to the MORGAM study, in Poland, smoking is less prevalent among women with low and secondary education than women with higher education, particularly in less urbanized areas [52]. A study indicated that in Eastern European countries, economic development and social and cultural processes associated with gender empowerment affect the differences in smoking between educated and uneducated women. It appears that the education related different patterns for smoking in Eastern European countries may interfere with the relationship between SED and mortality due to CSD in women, which may have influenced the lack of significance of the association [52, 53]. In our study, another important behavioral factor was BMI, which was associated with mortality due to CSD only in women. Although the prevalence of BMI varies by gender, obesity is more common in men below 45 years old, but at later ages above 45 years old, it predominates in women [54]. Some studies suggest that the risk of developing heart disease in women is increased by obesity coexisting with some classical CSD factors, i.e. diabetes, hypertension and hypercholesterolemia. Compared to men, these factors predispose women to heart disease to a greater extent [55].

It is worth noting the results obtained in assessing the association between SED and IHD mortality. A significant relationship was not found in univariate analysis, but the inclusion of additional explanatory variables nevertheless showed the existence of such a relationship. This may be related to the problem of omitted-variable bias: in the analysis ignoring the additional variables, the univariable model necessarily imputed their effect to the SED scale. By adding the omitted variables, the SED scale no longer assumes their partial effect, but reflects its “true” effect, which turns out to be statistically significant. Furthermore, this study confirmed the relationship between SED and mortality from IHD, with increased deprivation more strongly increasing mortality in women than in men (by 30.3% vs. 19.5% per SED scale unit). This significant result in both sexes may indicate similar risk factors for IHD, which may be explained by the fact that deprivation co-occurs with stress-related biological risk factors such as hypertension and diabetes [8, 18]. However, for the higher score in women, significant hormonal changes during menopause may play a role, which may also aggravate preexisting risk factors [56]. As in other studies [57, 58], we found the relationship between SED and mortality due to CeVD only in women. An explanation for this meaningful relationship may be psychosocial factors, which play a more significant role in women; for instance, depression may mediate the relationship between low SED and stroke [57]. However, the unclear result in the male population may be associated with a lower risk of stroke among unskilled manual workers compared with high-grade civil servants and executives [58].

Our results showed inequalities in mortality across sub-regions ranked by deprivation. The lowest CSD mortality was found in the less deprived sub-regions, which predominantly include large urban agglomerations and small cities. This observation may be associated with the fact that these sub-regions have access to different resources, including educational infrastructure, services, and job opportunities [59]. Furthermore, these sub-regions offer a more favorable environment, such as access to gyms and shops selling healthy foods, as well as health care services, which may contribute to better health outcomes in their residents [45, 60, 61]. The more deprived sub-regions are located in the eastern and northwest parts of Poland and are characterized by a low population density. These are considered less attractive to investors, which affects economic development. This confirms, among others, the fact that the areas of many sub-regions with more deprived sub-regions overlap with those areas in which national farm holdings had been liquidated and more harmful effects of the economic transformation are experienced. These areas lack support programs targeting social groups deprived of earlier forms of employment, and are consequently linked with poor health outcomes [26, 27].

Furthermore, the area encompassing sub-regions with a high deprivation in the western part of the country was associated with a more clear trend toward job migrations to work legally abroad. The migrations may exacerbate the optimal development of these areas due to the outflow of human capital and are also associated with a prevalence of social exclusion and poverty, leading altogether to health hazards [26, 62]. Some analyses indicate that the eastern and northwestern parts of the country have high total mortality due to CSD of the age group 25–64 years [63]. The issue of worse cardiovascular health in areas where national farm holdings were eliminated and areas with intensive labor migration is an interesting topic that needs further research.

For IHD, mortality was the highest in the less deprived sub-region group. In the case of CeVD, the highest mortality was found in middle sub-regions, which may suggest the occurrence of other specific factors that are not included in the analysis [64]. Indeed, for both IHD mortality and CeVD, positive relationship with the SED index has been confirmed using multivariable models that account for additional sources of mortality variation. It should also be emphasized that a rapid decrease in CSD mortality was noted in more deprived sub-regions with high SED index values. In the more deprived and middle group sub-regions, the percentage of DPP was higher compared to the less deprived sub-regions.

While the mortality due to CSD and CeVD was the lowest in the less deprived sub-regions, the reduction of mortality between 2010 and 2014 in these sub-regions was smaller compared to the sub-regions with the high SED index. This could be explained by the fact that in the best-developed—highly urbanized—sub-regions, mortality (due to better availability of cardiologic care services and invasive cardiology procedures) had been reduced in earlier years and the scope for improvement of cardiovascular health was narrower [65]. Simultaneously, modern prevention and therapeutic methods are increasingly becoming accessible in more deprived and middle sub-regions. For example, life-saving invasive cardiology procedures have been available after 2000 not only in academic centers but also in district hospitals, which might have resulted in reduced mortality even in smaller centers with a higher SED index. This is also supported by the results of other analyses [65], according to which the reduction of mortality due to CSD among people with higher education was particularly pronounced between 1991 and 1993 and 2001–2003, whereas during the period 2001–2003 and 2010–2012, the reduction was considerably lower. Noticeably, this mortality reduction was observed at a lower rate in people with low education compared to those with higher education.

Our results have shown that mortality from CSD, IHD and CeVD has decreased, and similar trends prevail in most European Union (EU) countries [1, 4, 5, 63]. It is also worth noting the progressive improvement in the health status of the Polish population, as evidenced by the systematically decreasing mortality rate of both younger and older people [63, 66]. At the same time, however, it must be said, against the background of the EU countries in the analyzed period 2010–2014, the mortality rates due to CSD in Poland were 60% higher compared to the average level for the EU, but already comparing to the old EU-15 countries were higher by 90%. For example, the level of mortality due to CSD in Poland is three times higher compared to France and the Netherlands. In contrast, against the background of the EU-13 countries that joined the EU after 2003, the level of mortality due to CSD in Poland is relatively low, i.e. the rates are 15% lower. Among Poland’s neighboring countries, the level of mortality due to CSD in Poland was 50% higher compared to Germany and similar to the level of mortality in the Czech Republic and Slovakia [67]. The existence of significant disparities in the level of mortality due to CSD between Poland and EU countries, especially the old EU-15, may indicate that there are opportunities for further mortality reduction. The stratification using terciles allowed to identify the residence-related deprivation, which may be necessary at intervention strategies and activities addressing improving cardiovascular health. In the policy process aimed to alleviate health inequalities, the activities should focus not only on poverty reduction throughout income redistribution. It is also critical ensuring equality of health opportunity in the entire population, through education, employment, improved working conditions and preventive care [68]. Decreasing health inequalities ought to be a political and social priority, given that they deteriorate economic productivity and the potential for sustainable and inclusive growth.

### Strengths and limitations

To our knowledge, this study is the first to use the SED index to assess the relationship with mortality due to CSD at the population level in Poland. The study was performed considering the whole population of the country. In the 66 sub-regions, a large variation was found in CSD mortality and SED. Furthermore, the sub-regions represented all the characteristics that are typical for Poland. The synthetic SED index enabled an approximate estimation of the singular variables (education, structure in employment, salary, unemployment, and poverty). A database concerning sub-regions defined based on NUTS-3 classification, which is used in the EU member states, was used for the first time in this study [32]. A unique strength of this study was the comparison of mortality due to CSD and the time-related changes using the DPP index in three different environments regarding deprivation level, thus, contrary to other studies reporting mortality trends in administrative areas of the country [65]. Our results showed a potent effect of health inequalities. Therefore, they may contribute to limited literature field dealing with the associations between area-related deprivation and mortality from CSD in Central and Eastern European countries [21, 22]. Similar and comparable socioeconomic levels in specific countries of this region may facilitate cross-comparisons and allow consistently conducting research across the populations.

The results of the study should be interpreted in light of certain limitations. The ecological design does not allow addressing the causality of the relationships. As sub-regions were considered as statistical units in this study, instead of individual persons, it was possible to investigate the inequalities between them, while inequalities within them remain unexplored. Epidemiological analyses for geographical areas lead to the best results when statistical units are populations of small size [69, 70]. It allows for better homogeneity and decreases the problem of averaging of different populations within a single geographical area. Such averaging results in attenuation of studied effects in statistical models. In this paper, population sizes in sub-regions were relatively large, so it was expectable that real effects might be significantly stronger and so their detection could be difficult, if not impossible. However, we were able to confirm statistically significant associations of the SED index with mortality from each of the three causes of death analyzed, although not always for both sexes. One explanation for the difficulty in demonstrating these relationships in each case may be the aforementioned high level data aggregation at the sub-regional level and the blurring of the relationships studied. In contrast to mortality and SED, information on covariates such as education, smoking, and BMI was based on one-point observation (Census 2011, Social Diagnosis Survey 2011), which was the only available for the studied sub-regions within the study period. It is unlikely that these characteristics changed much within the observation time. The relationship between SED and mortality due to CSD could be confounded by the sub-region differences in the exposure to the other uncontrolled factors; therefore, residual confounding should be considered. For example, we did not utilize stress, an important variable, because of the limited accessibility of such data. However, stress is regarded as a significant mediator linking the associations between deprivation and mortality due to cardiovascular diseases [71]. Noticeably, some components of the SED (e.g. percent of people on social support due to poverty) may have, at least partly, reflected chronic stress [18]. Thus, the role of stress should be investigated in future research. Another limitation of the study is the quality of the data on deaths associated with the differences in the reliability of death-cause coding by physicians who filled out death certificates. Our results showed that the highest mortality due to IHD was observed in the less deprived (most urbanized) sub-regions. This may be partially explained by the discrepancies in death certification encoding described earlier in Poland [72]. Such an issue was even reported in countries with highly advanced health information systems [73]. However, territorial differences in death-cause coding could rather contribute to the greater impact of random variability on our results, and it is less likely that the occurrence of a systematic error would explain the observed relationships. The latter is supported by the overall consistency of relationships found for IHD, CeVD, and CSD.

## Conclusions

Significant differences in mortality changes due to CSD in Poland could be observed in relation to socioeconomic deprivation, resulting in reduced health inequality. To reduce CSD mortality, more comprehensive preventive measures, including approaches addressing the socioeconomic factors, mainly poverty, education and employment, are needed, particularly in less urbanized areas. Further, well-designed studies should consider the contribution of psychosocial factors and their effect on cardiovascular health.

## Supplementary Information

**Additional file 1:** **Supplemental Fig. 1.** Geographic distribution of the SED index components (averaged data over the years 2010–2014) in 66 sub-regions of Poland.

## Data Availability

The datasets analyzed during the current studies are publicly available in Central Statistical Office, Poland (https://stat.gov.pl).

## Abbreviations

BMI: body mass index
CeVD: cerebrovascular diseases
CI: confidence interval
CSD: circulatory system diseases
DPP: deaths prevented or postponed
EU: European Union
GEE: generalized estimating eqs.
ICD: International Statistical Classification of Diseases and Related Health Problems
IHD: ischemic heart disease
NUTS: Nomenclature des Unités Territoriales Statistiques
SED: socioeconomic deprivation
SEP: socioeconomic position
